# First Detection of the CTXM-15 Producing *Escherichia coli* O25-ST131 Pandemic Clone in Ecuador

**DOI:** 10.3390/pathogens7020042

**Published:** 2018-04-16

**Authors:** Carlos Chiluisa-Guacho, Javier Escobar-Perez, Marise Dutra-Asensi

**Affiliations:** 1 Instituto Oswaldo Cruz, Fundação Oswaldo Cruz, Laboratório de Pesquisa em Infecção Hospitalar 4365, Rio de Janeiro 21040-900, Brasil; marise@ioc.fiocruz.br; 2 Instituto Nacional de Investigación en Salud Pública, INSPI “Dr. Leopoldo Izquieta Pérez”, Sede Zonal, 1501 Tena, Ecuador; 3 Laboratorio de Genética Molecular Bacteriana, Universidad El Bosque, 999076 Bogotá, Colombia; javiesco21@yahoo.com

**Keywords:** *Escherichia coli*, resistance, ST131, ESBL, MLST

## Abstract

Our aim was identify of the pandemic B2-ST131 *Escherichia coli* clone by to the Institute Pasteur and Achtman scheme, and investigate the resistance profile phenotypic-genotypic, with identification of class 1 integron. Of thirty-five ESBL-producing isolates recovered of patients with diagnosis of urinary tract infections (UTI), six *E. coli* strains serotype O25 were identified with resistance antimicrobial to several groups of antibiotics such as broad-spectrum cephalosporins, fluoroquinolones and aminoglycosides, harboring *blaSHV, blaCTX-M* genes in all isolates and *blaTEM* in two isolates. Sequencing of *blaCTX-M* revealed CTX-M-15 in all strains. The PMQR *aac(6´)-Ib-cr* and *qnrB19* genes were presented in five and four isolates respectively, AMEs genes *aac(6´)-Ib* and *aac(3)-IIa* were presented in strain amikacin-gentamicin-resistant. Sequencing of the variable regions of the class 1 integron revealed *dfrA* and *aadA* genes cassette. The analysis of multilocus sequence typing (MLST) confirms the presence of the pandemic B2-ST131 *E. coli* clone by Achtman scheme in all ST43 isolates obtained by of the Institute Pasteur scheme. The results presented herein, reveal the presence of B2-ST131 *E. coli* clone in Ecuador, disseminated in hospitals and community settings.

A multi-resistant clonal group of *Escherichia coli* ESBL-producing isolates, O25b:H4/ST131, was identified in 2008 as a major clone linked to CTX-M-15. Since then, it has also been strongly associated with fluoroquinolone resistance and co-resistance to aminoglycosides and trimethoprim-sulfamethoxazole [1]. This clone presents multiple antimicrobial resistance patterns, including resistance genes such as *bla*_CTX-M-15_, *bla*_TEM-1_, *bla*_OXA-1_, *aac(6*’*)-Ib-cr, qnrB*, and *aac(3)-II*, which are mainly located at plasmids belonging to the IncF group [2]. Currently, the B2-ST131 *Escherichia coli* clone is spread worldwide among humans, but it is also frequently recovered from livestock, companion animals, and food [3]. The present report describes the first detection of the *Escherichia coli* B2-ST131 clone isolated from patients diagnosed with urinary tract infections (UTI) in Quito-Ecuador.

We investigated 35 non-duplicate *Escherichia coli* ESBL-producing isolates, recovered from patients with nosocomial/healthcare-acquired or community acquired UTIs, in the metropolitan area of Quito, Ecuador. Isolated between July and December 2012, six isolates of *Escherichia coli* serotype O25 were identified with resistance to several groups of antibiotics, and the molecular characteristics of resistance in these isolates were studied. The antimicrobial susceptibility profile was determined via the agar diffusion method in accordance with the 2015 Clinical & Laboratory Standards Institute, CLSI Guidelines. Following species identification and antimicrobial susceptibility testing, isolates were screened for ESBL-producing phenotypes using double-disc synergy. The minimum inhibitory concentrations (MICs) of ciprofloxacin, gentamicin, and cefotaxime were determined using Etest strips following the manufacturer’s recommendations. Polymerase chain reaction (PCR) testing was performed in order to determine the phylogenetic relationship of these isolates, as per the methodology used by Clermont [4], namely, the detection of the class 1 integrons and the β-lactamases genes *bla*_SHV_, *bla*_TEM_, *bla*_CTX-M_, *bla*_AMPC_; PMQR genes *qnrA*, *qnrB*, *qnrS,* and *aac(6*’*)-Ib-cr*; AMEs genes *aac(3)-IIa*, *aac(6*’*)-Ib,* and *ant(2”)-Ia*; and 16S rRNA methylase genes *armA, rmtA, rmtB, rmtC, rmtD,* and *npmA*. Amplification products were purified and sequenced in a 3730 DNA Analyzer (Applied Biosystems, Foster City, CA, USA), at the PDTIS-IOC DNA Sequencing Platform. Sequences were compared to those in the GenBank database. Multi-locus sequence typing (MLST), were determined using the platform for *E. coli* MLST maintained at the Institute Pasteur, Paris, France, (http://bigsdb.pasteur.fr/ecoli/ecoli.html) as well as the Achtman multi-locus sequence typing scheme (http://mlst.warwick.ac.uk/mlst/dbs/Ecoli/documents/primersColi_html).

These six isolates of *E. coli* obtained from patients with UTIs, of which four were community-acquired and two were nosocomial, showed great resistance to broad-spectrum cephalosporins, fluoroquinolones, and aminoglycosides, confirmed by MICs. In addition, we detected co-resistance to various groups of antibiotics (Table 1). Sequence type 131 *Escherichia coli* clones were found to possess *bla*_SHV_ and *bla*_CTX-M_ in all isolates, with *bla*_TEM_ present in two isolates. *bla*_AMPC_ genes were not detected. Sequencing of *bla*_CTX-M_ revealed CTX-M-15 in all isolates. The PMQR *aac(6*’*)-Ib-cr* and *qnrB19* genes were present in five and four isolates, respectively. AMEs genes *aac(6*’*)-Ib* and *aac(3)-IIa* were present in amikacin-gentamicin-resistant strains. *16S rRNA methylase* genes were not detected and sequencing of the variable regions of the class 1 integron presented *dfrA* and *aadA* genes in five isolates. The MICs of cefotaxime ranged from 2 to >32 µg/mL, those of gentamicin ranged from 8 to >16 µg/mL, and those of ciprofloxacin ranged from 2 to >32 µg/mL. The multi-locus sequence typing scheme, designated according to the Institute Pasteur, revealed the clone B2-ST43, and the Achtman scheme confirmed the presence of the B2-ST131 *E. coli* clone in all isolates. Several other sequence types were present (ST2, ST160, ST365, ST173, ST21, ST477, ST472, and ST38).

The B2-ST131 *E. coli* clone has recently emerged, spreading out throughout the world, and is responsible for community and hospital-acquired urinary tract and bloodstream infections [1,2,3]. This clone represents a growing public health concern, primarily due to its resistance to various antimicrobial agents and its possession of high numbers of virulence factors, increasing morbidity and mortality [3]. The presence of several resistance genes in this clone has given it a broad antibiotic resistance profile, mainly against β-lactam antibiotics, aminoglycosides, and fluoroquinolones, all of which are widely used in the treatment of urinary tract infections. This study highlights the resistance of one strain to aminoglycosides and trimethoprim/sulphametoxazole, which lacks AMEs and *dfrA*/*aadA* genes, according to previous studies (Table 1). Epidemiological studies using pulsed-field gel electrophoresis (PFGE) have demonstrated that *E. coli* ST131 strains exhibit diverse pulsotypes [2]. In this research, we observed two clones with 94.1% similarity and one clone with less than 85% similarity (Figure 1). Our analysis of multi-locus sequence typing confirmed the presence of the pandemic B2-ST131 *E. coli* clone by the Achtman scheme in all ST43 isolates obtained by the Institute Pasteur scheme, in agreement with previous studies [5]. As such, it is important to maintain a surveillance system for the identification of clones representing a public health threat, particularly those that occur outside of the hospital environment.

## Figures and Tables

**Figure 1:**
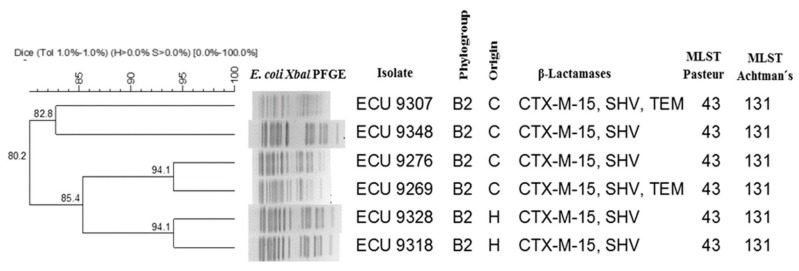
Dendrogram of pulsed-field gel electrophoresis (PFGE) patterns showing the genetic relatedness of the uropathogenic *Escherichia Coli* ST131 clone. C, community; H, Hospital.

**Table 1:** Phenotypic and genotypic features of uropathogenic *E. coli* ST131.

Isolate	PG	Phenotypic Profile	Genetic Profile			
		Co-Resistance ^a^	β-Lactamases	PMQR	AMEs	Integron 1
ECU9269	B2	CTX-CAZ-ATM-AM-CF-AMC-CIP-NOR	CTX-M-15, SHV, TEM	*aac(6’)-Ib-cr*		
ECU9276	B2	CTX-ATM-AM-CF-AMC SXT-AK-CIP-NOR	CTX-M-15, SHV	*aac(6’)-Ib-cr*	*aac(6’)-Ib*	*dfrA17, aadA5*
ECU9307	B2	CTX-FEP-CAZ-ATM-AM-CF-AMC-SXT-CN-CIP-NOR	CTX-M-15, SHV, TEM	*aac(6’)-Ib-cr/qnrB19*	*aac(6’)-Ib/aac(3)-IIa*	*dfrA12, aadA2*
ECU9318	B2	CTX-AM-CF-AMC-SXT-AK- CIP-NOR	CTX-M-15, SHV	*qnrB19*	*aac(6’)-Ib*	*dfrA17, aadA5*
ECU9328	B2	CTX-AM-CF-AMC-SXT-CIP-NOR	CTX-M-15, SHV	*aac(6’)-Ib-cr/qnrB19*		*dfrA17, aadA5*
ECU9348	B2	CTX-FEP-CAZ-ATM-AM-CF-AMC-SXT-CN-CIP-NOR	CTX-M-15, SHV	*aac(6’)-Ib-cr/qnrB19*	*aac(6’)-Ib/aac(3)-IIa*	*dfrA17, aadA5*

AK, amikacin; CN, gentamicin; SXT, trimethoprim–sulphamethoxazole; CIP, ciprofloxacin; NOR, norfloxacin; AM, ampicillin; ATM, aztreonam; CF, cefalothin; FOX, cefoxitin; CTX, cefotaxime; CAZ, ceftazidime; FEP, cefepime; AMC, amoxicillin-clavulanic acid; PMQR, plasmid-mediated quinolone resistance determinant; AMEs, aminoglycoside-modifying enzymes; PG, phylogroup. **^a^** Antimicrobial non-susceptibility (i.e., resistant or intermediate).
